# Enhancing the Resistance of Soybean Plants to Waterlogging Stress Using Gamma-Irradiated *Priestia megaterium*

**DOI:** 10.3390/microorganisms14102145

**Published:** 2026-09-23

**Authors:** Sin-Tian Wang, Lih-Geeng Chen, Hung-Yu Shu, Shih-Yin Lu, Yu-Cheng Chen, Min-Chang Su, Shao-Hung Wang

**Affiliations:** 1 Department of Medical Laboratory Science and Biotechnology, College of Medicine, National Cheng Kung University, Tainan 701, Taiwan; 2 Department of Microbiology, Immunology and Biopharmaceuticals, National Chiayi University, Chiayi 600, Taiwan; lgchen@mail.ncyu.edu.tw (L.-G.C.);; 3 Department of Bioscience Technology, Chang Jung Christian University, Tainan 711, Taiwan; 4 Department of Isotope Application Research, National Atomic Research Institute, Taoyuan 325, Taiwan; mc.su@nari.org.tw

**Keywords:** plant growth-promoting rhizobacteria (PGPR), waterlogging stress, *Priestia megaterium*, gamma-ray irradiation

## Abstract

The rhizosphere of *Fabaceae* contains plant growth-promoting rhizobacteria (PGPR) that regulate phytohormone balance, nutrient acquisition, and biofilm formation, thereby contributing to plant development and stress tolerance. Although Indole-3-acetic acid (IAA) and exopolysaccharides (EPS) are well-established PGPR-associated factors, their combined contributions to waterlogging tolerance in soybean remain unclear. In this study, *Bacillus*-like PGPR were isolated from the rhizosphere of soybean plants cultivated in field soil, and *Priestia megaterium* and *P. aryabhattai* were identified among *Bacillus*-like bacteria based on strong EPS production, a PGPR-associated trait. The *P. megaterium* isolate S135-A1-2 was subjected to gamma-ray irradiation to generate mutants, from which mutants #6 (increased EPS), #8 (increased IAA), and #12 (increased EPS and IAA) were selected for further analysis. Mutant #8 produced 4.48 μg/mL IAA, a 3.6-fold increase over the parental strain, and protease and siderophore activities were also evaluated as additional PGPR-associated traits. Their plant growth-promoting traits were evaluated using pot assays under waterlogging conditions. Inoculation with mutants #6 and #8 increased lateral root development and root biomass relative to the parental strain, whereas mutant #12 showed a distinct response pattern associated with dual-trait enhancement under waterlogging stress. After 7 days of waterlogging, mutant #12 showed higher chlorophyll-related values than mutants #6 and #8. These results indicate that gamma-irradiated *P. megaterium* mutants can differentially modify soybean root system responses to flooding stress, supporting gamma-ray irradiation as a useful approach for developing PGPR with improved functions for agricultural applications under waterlogging stresses.

## 1. Introduction

Soybeans are highly valued as a major food crop worldwide due to their high protein and oil content and the presence of health-promoting compounds such as isoflavones and phenolic compounds. These bioactive compounds have been associated with reduced risks of cardiovascular disease, osteoporosis, and certain cancers [1]. However, soybean development and productivity are heavily constrained by abiotic environmental factors, including excessive rainfall and drought. Due to climate change caused by global warming, Taiwan experiences highly concentrated rainfall during the rainy season and marked disparities in precipitation between wet and dry periods. Soybeans are therefore particularly susceptible to waterlogging, which restricts oxygen diffusion in the rhizosphere and causes root hypoxia, thereby impairing root respiration, nutrient uptake, root system development, and plant growth [2].

Rhizosphere-associated plant growth-promoting rhizobacteria (PGPR) support plant development by modulating hormone levels, including 1-aminocyclopropane-1-carboxylate (ACC) metabolism, reducing ethylene production, adjusting hormonal balance, stimulating antioxidant enzyme synthesis, enhancing mineral uptake, and promoting exopolysaccharide (EPS) production to alleviate stress [3,4]. Some PGPRs have been widely used to enhance the stress tolerance of soybean plants, including *Acinetobacter*, *Alcaligenes*, *Arthrobacter*, *Azospirillium*, *Azotobacter*, *Bacillus*, *Beijerinckia*, *Burkholderia*, *Enterobacter*, *Erwinia*, *Flavobacterium*, *Pseudomonas*, and *Serratia*, among others. They stimulate plant growth through the production of amino acids, IAA, gibberellins, and exopolysaccharides, resulting in improved root growth, enhanced water and nutrient uptake, and the formation of rhizobium–soybean interaction sites [5].

The Indole-3-acetic acid (IAA) promotes short-term cell elongation and long-term cell division and differentiation in plants [3], particularly enhancing root development in soybeans. It has been reported that IAA produced by PGPR is a crucial signaling molecule in plant–microbe interactions, directly contributing to enhanced plant growth [6]. Under waterlogging IAA secreted by PGPRs can increase the root surface area-to-volume ratio, thereby improving root system development and enhancing nutrient and water uptake [7,8].

*Bacillus* spp. have been extensively investigated for their biocontrol, plant growth hormone synthesis, and improvement of rhizotrophic conditions to enhance soybean plant growth, including *B. amyloliquefaciens*, *B. mojavensis*, *B. subtilis*, and *B. velezensis* [9]. The hydrophobic exopolysaccharide (EPS) is secreted and associated with the *bslA* gene-harboring *B. subtilis* [10]. *Bacillus subtilis* secretes exopolysaccharide (EPS) and the amyloid-forming protein TasA to form biofilms, thereby enabling stable colonization of plant roots and promoting plant growth [9,11]. It has been demonstrated that *B. megaterium* promotes soybean growth under environmental stress through PGPR characteristics, including enhanced IAA and gibberellin production and phosphate solubilization [12]. Recent studies further indicate that *Priestia megaterium* (formerly *B. megaterium*) enhances plant growth through nutrient mobilization and stress-responsive root colonization [9,13].

Some studies have indicated that IAA and EPS play critical roles in soybean growth and in resistance to waterlogging stress. In contrast, siderophore production and protease activity may contribute to waterlogging tolerance more indirectly: siderophores can improve iron acquisition and microbial competitiveness, whereas protease activity may promote nutrient cycling and suppress soil-borne pathogens. Gamma-ray irradiation has been used as a mutagen in mutation-breeding programs to generate genetic variation and select variants with altered agronomic traits [14]. In our previous study, gamma-ray irradiation was applied to *Rhizopus oligosporus* to induce large-scale random genetic variation, and mutant strains with advantageous enzyme activities, including protease, lipase, and β-glucosidase activities, were successfully identified [15]. Moreover, gamma-irradiated *Bacillus* mutant candidates with enhanced traits have been obtained at doses ranging from 100 to 1000 Gy [16,17]. Herein, a dose of 500 Gy was applied to induce mutagenesis. In this research, we hypothesized that gamma-irradiated mutants with increased IAA and/or EPS production would improve soybean root development and waterlogging tolerance compared to the parental strain. We screened various potential PGPR strains, along with their gamma-irradiated mutants, to evaluate their capacities for IAA and EPS production. Strains exhibiting superior IAA and EPS production were subsequently inoculated into the soil to evaluate their effectiveness in mitigating waterlogging stress using pot culture assays.

## 2. Materials and Methods

### 2.1. Bacillus–like PGPR Candidates Isolated from Soybean Roots

Nine batches of rhizosphere soil samples were collected from a soybean experimental field. Each soil sample (1 g) was mixed with 5 mL of Luria–Bertani (LB) broth. After allowing the mixture to settle for 10 min, the supernatant was collected and incubated in a water bath at 80 °C for 10 min to kill vegetative cells. Spore-forming bacteria were isolated from the supernatant using the Tryptone Soy agar (TSA; BD DIFCO) plates containing cycloheximide (50 mg/L). Isolates were cultured on *Bacillus* ChromoSelect agar (Sigma-Aldrich), Pikovskaya’s agar (HiMedia Laboratories), and blood agar plates (BAP; Creative Life Science, New Taipei City, Taiwan) at 25 °C for 1 day to assess colony morphology, phosphate solubilization, and hemolysis, respectively. Antibacterial activity was assessed by spot-inoculating candidate strains onto semi-solid agar containing *E. coli* and observing clear inhibition zones after incubation at 25 °C for 1 day.

### 2.2. Molecular Identification of PGPR Candidates

A single colony was inoculated into Tryptone Soy broth (TSB; BD DIFCO) for 12 h. Genomic DNA was purified, and the 16S rRNA gene was PCR-amplified using primers 27f and 1492r [18]. The PCR product was purified and sequenced. The bacterial species were determined with the National Center for Biotechnology Information (NCBI) nucleotide collection (nr/nt) database using the web-based BLASTn platform (accessed in 2025).

### 2.3. Generation of PGPR Mutants by Gamma-Ray Irradiation

A single colony of the PGPR candidate was inoculated into TSB for 12 h, and the freshly cultured broth was dispensed onto sterile 0.22-μm filter membranes. The paraffin-sealed membranes were transported to the National Atomic Research Institute, where PGPR mutants were generated by gamma-ray irradiation. Gamma-ray irradiation at 500 Gy, which resulted in approximately 98% mortality, was applied. The surviving strains were collected in 1 mL of LB broth and streaked onto TSA, followed by incubation at 25 °C for 1 day. Thirty gamma-irradiated mutants were randomly selected from the parental PGPR strain.

### 2.4. Polysaccharide String Test

The candidates were streaked on TSA containing 2% dextrose and incubated at 25 °C until the colony diameter exceeded 0.1 cm. The strings were then drawn from the colony using sterile toothpicks, and the difference between the extent of string formation and the colony diameter was calculated.

### 2.5. The Quantification of Indole-3-Acetic Acid (IAA)

The Salkowski reagent is a mixture of 0.5 M ferric chloride (FeCl_3_) and 35% perchloric acid (HClO_4_). IAA standards were prepared, and 500 μL of each standard solution was mixed with Salkowski reagent and incubated at 25 °C in the dark for 30 min. The OD_530_ was measured to establish the IAA calibration curve. A single colony of wild-type candidate strains was inoculated into TSB for 12 h. The culture was incubated in a 24-well plate containing 1 mL of Winogradsky’s medium at 25 °C for 7 days. After centrifugation, 250 μL of supernatant was mixed with 500 μL of Salkowski reagent and incubated at room temperature for 30 min. A volume of 200 μL was then transferred to a 96-well plate, and OD_530_ was measured to calculate IAA production [19].

### 2.6. Evaluation of the Exopolysaccharides

A single colony of the mutant strains was inoculated into 1 mL of TSB and cultured overnight. The mutant strains were streaked on Congo Red agar plates and incubated at 25 °C for 2 days in the dark. Colonies displaying a dark brown phenotype were selected and subcultured in TSB at 37 °C for 48 h. For EPS extraction, bacterial cells were removed by centrifugation at 12,000× *g* for 30 min at 4 °C. The culture supernatant (10 mL) was transferred to fresh tubes and mixed with two volumes of ethanol, followed by incubation at 4 °C for 48 h. Precipitated EPS was collected by centrifugation at 15,000× *g* for 30 min, washed twice with sterile water, dried at 65 °C, and resuspended in 1 mL sterile water. Total EPS was quantified using the phenol–sulfuric acid assay at OD_492_ with reference to a standard curve. EPS yield was expressed as μg/mL of culture supernatant.

### 2.7. Protease Activity Characterization

Single colonies of each mutant strain were suspended in sterile distilled water, and the cell density was adjusted to OD_600_ = 0.1 ± 0.01. The standardized bacterial suspensions were then inoculated onto casein agar plates and incubated at 37 °C for 24 h to observe the formation of proteolytic clear zones. Protease activity was expressed as the enzyme index (EI), calculated as the diameter of the proteolytic clear zone divided by the colony diameter.

### 2.8. Siderophore Activity Assessment

Bacterial strains were streaked on TSA plates and incubated at 37 °C for 1 day. Melted Chrome azurol S (CAS) agar was then overlaid onto the plates containing bacterial colonies. Following a 4-day incubation period at 30 °C, siderophore activity was verified by observing distinct orange/yellow halo zones surrounding the colonies [20].

### 2.9. Soybean Plant Stress Tolerance Analysis

The stress tolerance of soybean plants inoculated with PGPR candidates or their gamma-irradiated mutants was investigated under waterlogging conditions. In detail, soybean seeds were soaked in 2.5% (*v*/*v*) sodium hypochlorite for 15 min and washed with sterilized water 3 times, inoculated in a plastic culture container containing peat, and cultured at 26 °C for 3 days in the dark to grow seedlings. Uniform seedlings were randomly assigned to the non-inoculated control, wild-type, and gamma-irradiated mutant treatments. Each treatment included four independent pots, each containing one seedling. Pots were randomly positioned in the growth chamber during the experiment. Bacterial cultures were adjusted to 10^7^ CFU/mL (total volume 20 mL), and soybean seedlings were immersed in the suspension for 20 min. The soybean seedlings were transferred to a plastic pot containing peat soil and inoculated additionally with 1 mL of bacterial culture and incubated in a plant incubator for 5 days at 26 °C, humidity 50–60%, and light 8000 lux. For the waterlogging treatment, each soybean pot was placed inside a larger beaker, and the soil was inundated with sterile distilled water to a depth of 5 cm, using an additional empty pot to maintain the water level above the soil surface. The 26 °C incubator was programmed for a 16/8-h day-night cycle, and measurements and analysis of stem/leaf fresh dry weight, stem length, main/lateral root length, total/main/lateral root fresh dry weight, and chlorophyll content were conducted following a 7-day incubation period. Chlorophyll and carotenoids were extracted from 0.01 g fresh leaf tissue in 1 mL dimethyl sulfoxide at 65 °C for 24 h, and absorbance was measured at 480, 645, and 663 nm; pigment contents were calculated [21,22].

### 2.10. Statistical Analysis

Data are presented as mean ± SD. For each experiment, differences among treatments were evaluated by one-way analysis of variance (ANOVA) with Prism (GraphPad software, version 10.4.2), followed by Tukey’s multiple-comparison test. A value of *p* < 0.05 was considered statistically significant. For the waterlogging pot assay, each treatment included four independent pots, with one soybean seedling per pot (n = 4).

## 3. Results

### 3.1. Isolation, Screening, and Identification of Soybean PGPR Strains

To select endospore-forming bacteria by heat treatment, the 26 *Bacillus*-like strains were isolated from the soil collected from a soybean experimental field. For the first screening step, the isolates were excluded when exhibiting some phenotypes, such as human pathogens indicated by *Bacillus* ChromoSelect agar or hemolytic activity determined by Blood TSA plate, and low phosphate-solubility activity identified by Pikovskaya’s agar. In the second screening step, nine potential candidates showing phosphate-solubilizing activity and non-hemolytic properties were evaluated for polysaccharide production capacity. Finally, four potential PGPR candidates with superior polysaccharide production were selected using the polysaccharide string test, including strains S029-A1-3, S109-A-1-2, S109-A Lg-2, and S135-A1-2 (Figure 1A).

The candidate PGPR strains were identified by 16S rRNA gene sequence analysis using the National Center for Biotechnology Information (NCBI) nucleotide database, and their taxonomic assignment followed the reclassification of the *Megaterium* clade into the genus *Priestia* [23]. The results showed high similarity to *Priestia* species: S029-A1-3 (99.93% to *Priestia aryabhattai* strain fwzb7), S109-A-1-2 (100% to *Priestia megaterium* strain OO-7), S109-A Lg-2 (99.93% to *Priestia megaterium* DSM 31), and S135-A1-2 (100% to *Priestia megaterium* strain A-2). In addition, the four selected candidates exhibited casein-degrading activity (Figure 1C) but did not inhibit *Escherichia coli* growth (Figure 1B). The ranking of casein activity among the strains was S029-A1-3, S109-A-1-2, S109-A Lg-2, and S135-A1-2 (Figure 1C), indicating that all four wild-type isolates are plant growth-promoting rhizobacteria (PGPR).

### 3.2. Quantification of IAA Secreted by the Selected Bacteria

The IAA production of the four PGPR candidates and their gamma-irradiated mutants was evaluated using Salkowski reagent and quantified based on the IAA standard curve. The four PGPR candidates were classified into two groups: a high-IAA production group comprising S029-A1-3 (IAA: 4.41 ± 0.59 μg/mL) and S109-A Lg-2 (IAA: 3.42 ± 0.39 μg/mL); and a low-IAA production group comprising S109-A-1-2 (IAA: 1.24 ± 0.14 μg/mL) and S135-A1-2 (IAA: 1.58 ± 0.37 μg/mL) (Figure 2). IAA production was significantly increased in several gamma-irradiated mutants compared with their corresponding parental strains, including the S109-A Lg-2 mutants (#9, #15), S109-A-1-2 mutants (#6, #7, #8, #9, #10, #12, #13, #16, #19, #25, #29), and S135-A1-2 mutants (#8, #12, #14, #17, #18, #21) (Figure 2B–D). In contrast, the gamma-irradiated mutants of S029-A1-3 did not exhibit increased IAA production relative to the parental strain (Figure 2A). Notably, S135-A1-2 mutant #8 produced 4.48 μg/mL of IAA, representing a 3.6-fold increase over the parental strain and outperforming all other tested PGPR candidates and their gamma-irradiated mutants (Figure 2D).

### 3.3. Evaluation of EPS Production, Protease Activity, and Siderophore Activity in the Gamma-Irradiated Mutants

Some PGPR characteristics were evaluated in the four PGPR candidates and their gamma-irradiated mutants. Exopolysaccharide (EPS) production was first estimated by the Congo Red color reaction (Figure 3A), which showed that several mutants displayed a darker brown-red color than their parental wild-type strains, indicating higher polysaccharide production. These included seven mutants of S029-A1-3 (#2, #3, #4, #5, #6, #10, #11), 17 mutants of S109-A-1-2 (#1, #2, #3, #4, #5, #6, #7, #8, #9, #10, #16, #19, #20, #25, #27, #28, and #29), only one mutant of S109-A Lg-2 (#10), and five mutants of S135-A1-2 (#4, #6, #12, #21, #30) (Figure 3A). Mutant #8 produced EPS at a level comparable to that of the parental S135-A1-2 strain (364.4 ± 23.7 vs. 353.3 ± 37.6 μg/mL of culture supernatant). In contrast, mutants #6 and #12 produced significantly higher EPS levels (588.3 ± 128.9 and 473.6 ± 25.4 μg/mL, respectively), indicating enhanced EPS production. Furthermore, protease enzyme activity (EI) was quantified in two PGPR wild-type strains from the low IAA-producing group, *P. megaterium* S109-A-1-2 and S135-A1-2, as well as in their gamma-irradiated mutants (Figure 3C). Both the parental wild-type PGPR strains and their gamma-irradiated mutants demonstrated robust proteolytic activity, with most mutagenized strains showing significantly higher proteolytic activity than their respective parental counterparts. Finally, the siderophore activity was examined in parental wild-type PGPR strains, *P. megaterium* S109-A-1-2 and S135-A1-2, as well as their gamma-irradiated mutants (Figure 3D).

### 3.4. Waterlogging Resistance of Soybean Plants Inoculated with Candidates

Examination of soybean responses to waterlogging stress was performed using the parental strain S135-A1-2 and the selected mutants S135-A1-2 #6 (high EPS), S135-A1-2 #8 (high IAA), and S135-A1-2 #12 (high EPS and IAA). Under waterlogging conditions, plants inoculated with mutants #6 and #8 showed greater lateral-root biomass than plants inoculated with the parental strain or the non-inoculated control, whereas responses of total-root and taproot biomass varied among treatments (Figure 4). In the normal control, the morphological characteristics of the soybean in the control group were similar to those of the soybean under waterlogging stress (Figure S1). Under waterlogging conditions, plants inoculated with mutants #6 (fresh weight: 0.99 ± 0.13 g; dry weight: 0.068 ± 0.007 g) and #8 (fresh weight: 0.92 ± 0.06 g; dry weight: 0.069 ± 0.004 g) developed more lateral roots than those inoculated with the parental strain (fresh weight: 0.63 ± 0.077 g; dry weight: 0.048 ± 0.005 g) or the non-inoculated control (fresh weight: 0.60 ± 0.15 g; dry weight: 0.045 ± 0.010 g). However, mutant #12 (fresh weight: 0.72 ± 0.075 g; dry weight: 0.055 ± 0.005 g) displayed a different response pattern, with root biomass not exceeding that of mutants #6 and #8.

Evaluation of chlorophyll-related traits under waterlogging stress also revealed differential responses among the inoculated treatments. Plants inoculated with mutant #12 had higher chlorophyll *b* content (0.32 ± 0.04 mg/g dry weight) than those inoculated with mutants #6 (0.25 ± 0.11 mg/g dry weight) and #8 (0.22 ± 0.05 mg/g dry weight) (Figure 5); however, these differences were not statistically significant. Overall, chlorophyll *b* content did not show a consistent increase across all inoculated groups compared with the non-inoculated control and wild-type strain.

**Figure 5:**
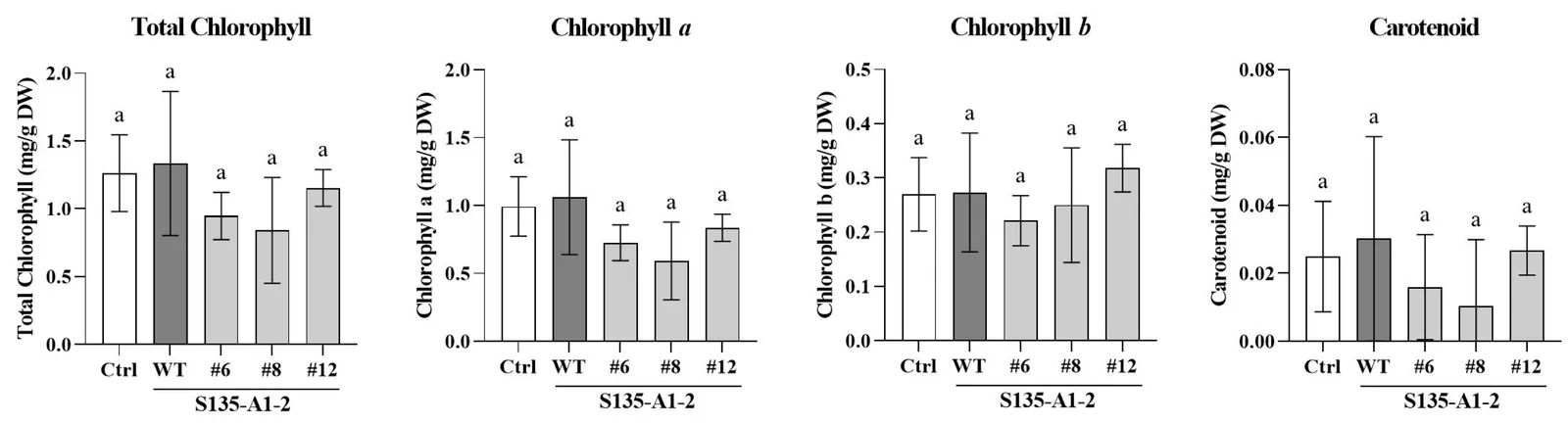
Chlorophyll and carotenoid contents in soybean plants inoculated with PGPR or mutants under waterlogging stress. Total chlorophyll, chlorophyll *a*, chlorophyll *b*, and carotenoids were measured in plants inoculated with wild-type S135-A1-2 (WT) and mutants #6, #8, and #12. Different lowercase letters indicate statistically significant differences among groups determined by one-way ANOVA followed by an appropriate Tukey’s multiple-comparison test (*p* < 0.05). Groups sharing at least one letter are not significantly different.

## 4. Discussion

Under waterlogging, the environment is oxygen-restricted for roots, resulting in suppressed root respiration and reduced root activity. The plant diminishes its light-capturing capacity, which ultimately decreases the photosynthetic rate, because the leaf stomata close, chlorophyll degrades, and leaf senescence and yellowing occur [24,25]. The plant coordinates hormone-induced defense responses to waterlogging stress, including the induction of adventitious roots and improvement of anaerobic respiration [24]. Moreover, the PGPR has also been reported in plants in response to waterlogging, including nutrient uptake and root development. The primary objective of this research was to isolate potential PGPR strains from the soybean rhizosphere and to generate gamma-irradiated mutants with enhanced functional traits. *Bacillus*-related bacteria are well known for synthesizing macromolecular complexes that support the rhizosphere in acquiring essential minerals and nutrients and producing PGPR-associated functional molecules, such as *B. megaterium* XTBG34 produces 2-pentylfuran, which significantly promotes growth in *Arabidopsis* plants [26]. In this study, the gamma-irradiated mutants derived from isolated PGPR displayed higher proteolytic activity than the parental strain. It suggests that PGPR isolates with elevated protease activity may potentially benefit host plants by improving the organic nutrient availability and uptake within the rhizosphere.

In waterlogged soils, iron (Fe^3+^) and manganese (Mn^4+^) are extensively reduced to highly toxic free Fe^2+^ and Mn^2+^ ions [27]. *Bacillus*-related PGPR produce exopolysaccharides and siderophores that modulate ion transport and enhance plant tolerance to environmental stress. Siderophores secreted by these bacteria bind ferric ions in the environment to form siderophore–Fe^3+^ complexes, which can subsequently release ferrous ions through reduction reactions under adverse conditions, thereby improving iron bioavailability [3,4]. The isolated parental wild-type PGPR strains and their derived mutants displayed distinct siderophore activity. Nevertheless, mutants with high siderophore activity may benefit host plants by enhancing resistance to pathogens through competitive iron sequestration.

Under waterlogging, soil fertility is rapidly lost due to leaching. The EPS secreted by PGPR can form a biofilm on the surface of soybean roots. The EPS molecular chains are rich in anionic functional groups, including hydroxyl (-OH) group and carboxyl (-COOH) group [28]. The EPS molecules may adsorb and chelate potentially phytotoxic reduced metal ions, thereby limiting their availability in the root-associated environment and potentially reducing oxidative damage [27]. In addition, EPS-mediated biofilm formation may buffer the root-associated microenvironment and reduce the impact of ion imbalance and oxidative stress during waterlogging. In our study, the mutants displayed varying abilities in EPS production. Soybean plants inoculated with mutant #6 had greater root biomass than the other groups. It indicates that the EPS produced by PGPR can protect soybean against waterlogging by blocking injury caused by heavy metal ions and hypoxia.

Previous studies have reported that inoculation with IAA-producing *Klebsiella variicola* improved adventitious root growth, waterlogging tolerance, and chlorophyll content in soybean [29,30]. At physiological concentrations of 1–10 μM (0.175 ~ 1.75 μg/mL), exogenous IAA acts as a key morphogenic trigger in soybean seedlings, primarily promoting lateral and adventitious root initiation while increasing the root surface-area-to-volume ratio [7,8]. However, higher concentrations approaching 10 μM can shift the hormonal balance by upregulating ACC synthase and triggering ethylene accumulation, which tends to inhibit primary root elongation [3,4,31,32]. Importantly, soybean root exudates contain amino acids, and tryptophan (Trp) released from roots may provide a substrate for Trp-dependent bacterial IAA biosynthesis in the rhizosphere. Thus, IAA production measured in culture medium may not fully predict the amount of IAA generated at the root surface, where the availability of plant-derived Trp and the extent of bacterial colonization can jointly determine local IAA exposure [33].

Previous research has indicated that PGPR strains, especially IAA-producing strains, can stimulate the growth of adventitious roots, thereby reducing ethylene content in plants and increasing chlorophyll levels [30]. In our study, it exhibited distinct PGPR-associated phenotypes characterized by IAA production of S135-A1-2 (IAA: 1.58 μg/mL) and their derived mutants #6 (IAA: 1.50 μg/mL), #8 (IAA: 4.48 μg/mL), and #12 (IAA: 2.78 μg/mL). The S135-A1-2 mutants #6 and #8, characterized by increased EPS and IAA production, respectively, enhanced lateral root development and root biomass under waterlogging, consistent with the role of IAA in promoting root growth and EPS in improving rhizosphere conditions. These results suggest that EPS should not be considered only as a stress-protective extracellular matrix. By promoting root attachment, microcolony formation, and biofilm development, EPS may contribute to the maintenance of bacterial populations at the root surface and efficiently access root-derived metabolites, including Trp. Under this framework, EPS may be a particularly relevant screening trait because it supports the initial establishment and persistence of the inoculant in the rhizosphere, whereas the local IAA output of colonized bacteria may subsequently be enhanced by plant-derived Trp.

In contrast, mutant #12, with simultaneous elevation of IAA and EPS, did not show the highest root biomass but exhibited higher chlorophyll-related values, although the differences were not statistically significant. This non-additive response suggests that the plant outcome is determined not simply by the absolute in vitro production of EPS and IAA, but by their spatial and temporal balance at the root–microbe interface. These findings suggest that EPS may influence the effective IAA exposure at the root surface, thereby modulating root growth and chlorophyll content under waterlogging stress. This highlights the importance of balancing PGPR traits to optimize both root and shoot responses under flooding.

In the waterlogging study, total chlorophyll decreased in soybean inoculated with the S135-A1-2 mutants #6 and #8 when compared to wild type, but not in soybean inoculated with the mutant #12. Under waterlogging, root hypoxia induces the accumulation and translocation of ethylene precursors to the leaves, triggering chlorophyll degradation [34,35]. Notably, the excessive IAA produced by the mutant #8 (4.48 μg/mL) might have further stimulated ACC synthase activity in hypoxic roots, leading to elevated stress-ethylene levels in the shoots. Consequently, the beneficial effects of PGPR on root system development and photosynthetic pigments were not necessarily concordant under severe flooding conditions.

Gamma-ray irradiation is expected to introduce random mutations by causing DNA damage, either directly or through oxidative stress. In this study, 500 Gy was selected to obtain mutant derivatives for screening, not to specifically stimulate IAA or EPS production. The enhanced IAA and EPS production observed in several mutants may have arisen from changes in genes involved in their synthesis or regulation. Genome sequencing will be useful for linking the altered phenotypes to specific mutations. The stability of these traits and their effects on soybean growth and waterlogging tolerance should also be examined in greenhouse experiments.

## 5. Conclusions

In this study, four PGPR candidates, including three *Priestia megaterium* isolates and one *P. aryabhattai* isolate, were obtained from the soybean rhizosphere. The *P. megaterium* isolate S135-A1-2 and its gamma-irradiated derivatives were subsequently evaluated for their effects on soybean responses under waterlogging conditions. The gamma-irradiated *P. megaterium* mutants with enhanced IAA and/or EPS production were associated with altered soybean responses under waterlogging conditions. The distinct phenotypic profiles of mutants #6, #8, and #12 highlight the potential to tailor PGPR traits to specific stress adaptation strategies. Gamma-ray irradiation thus represents a promising tool for generating functionally improved PGPR strains for sustainable agriculture under abiotic stress.

## Figures and Tables

**Figure 1:**
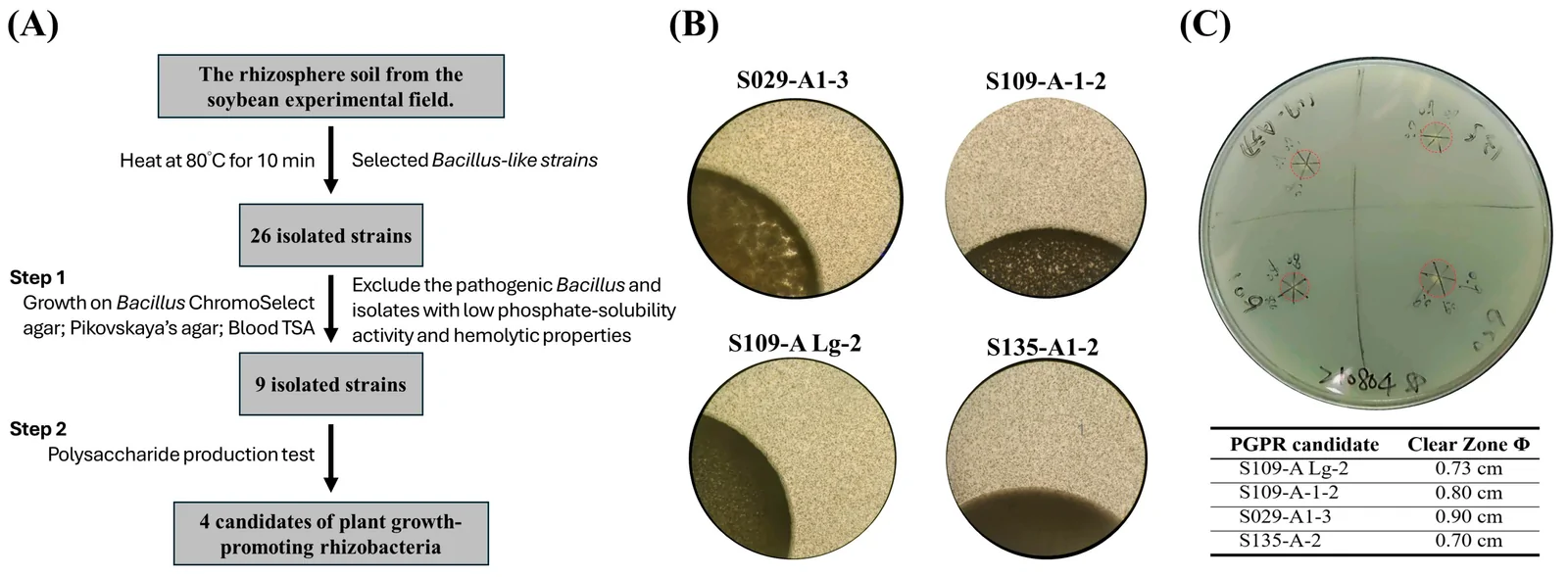
Potential PGPR candidates isolated from the soil of a soybean experimental field. (**A**) Flowchart of screening potential PGPR from soil samples. (**B**) Inhibition of *E. coli* by the candidates. (**C**) Proteolytic casein-clearing zones of the candidates.

**Figure 2:**
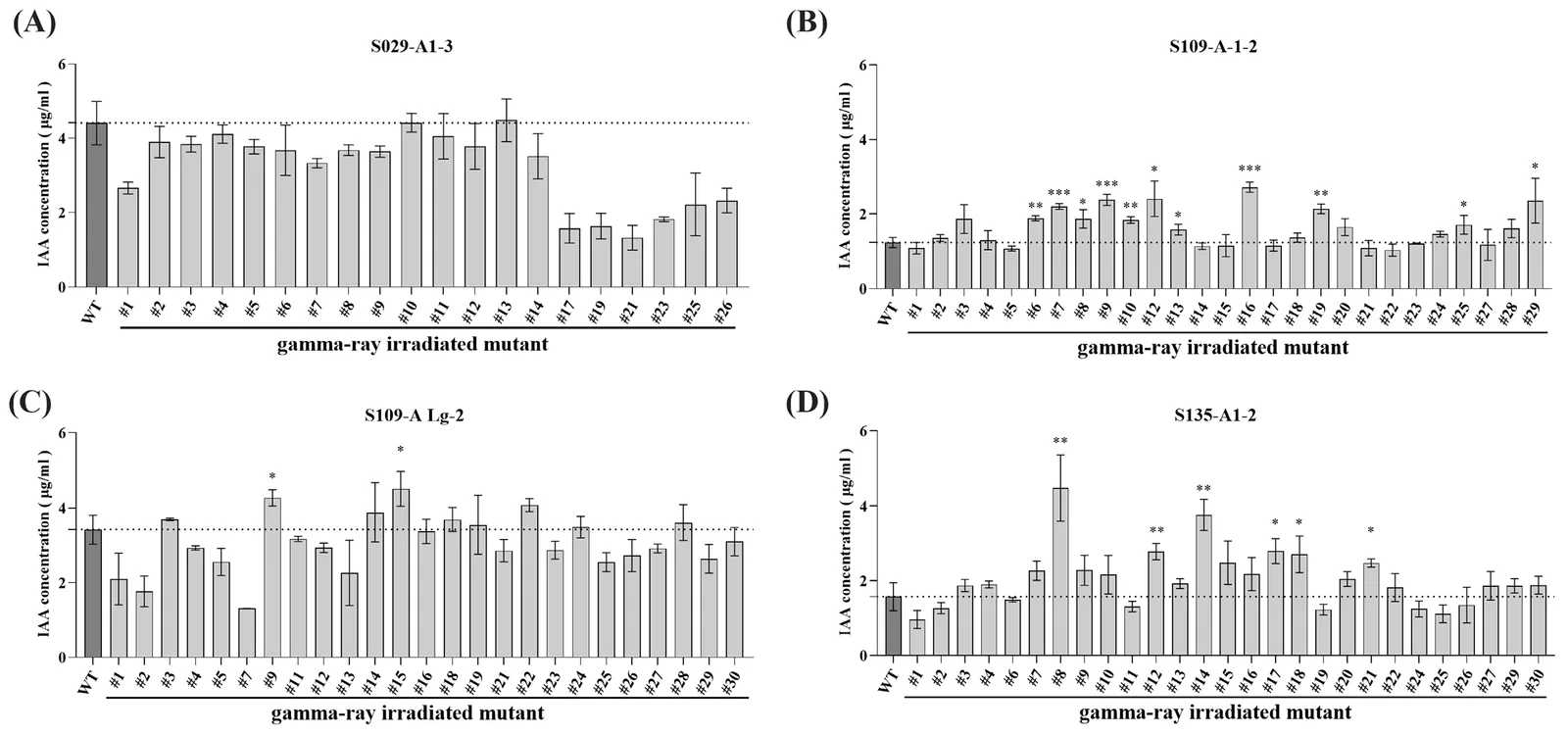
IAA production in PGPR candidates and gamma-irradiated mutants. Indole-3-acetic acid (IAA) was quantified in parental PGPR candidates and their mutants, including (**A**) S029-A1-3, (**B**) S109-A-1-2, (**C**) S109-A Lg-2, and (**D**) S135-A1-2. The dotted lines indicate the mean values for the wild-type isolates. Asterisks indicate significant differences compared with the parental wild-type strain, which served as the irradiation control. * *p* < 0.05, ** *p* < 0.01, *** *p* < 0.001.

**Figure 3:**
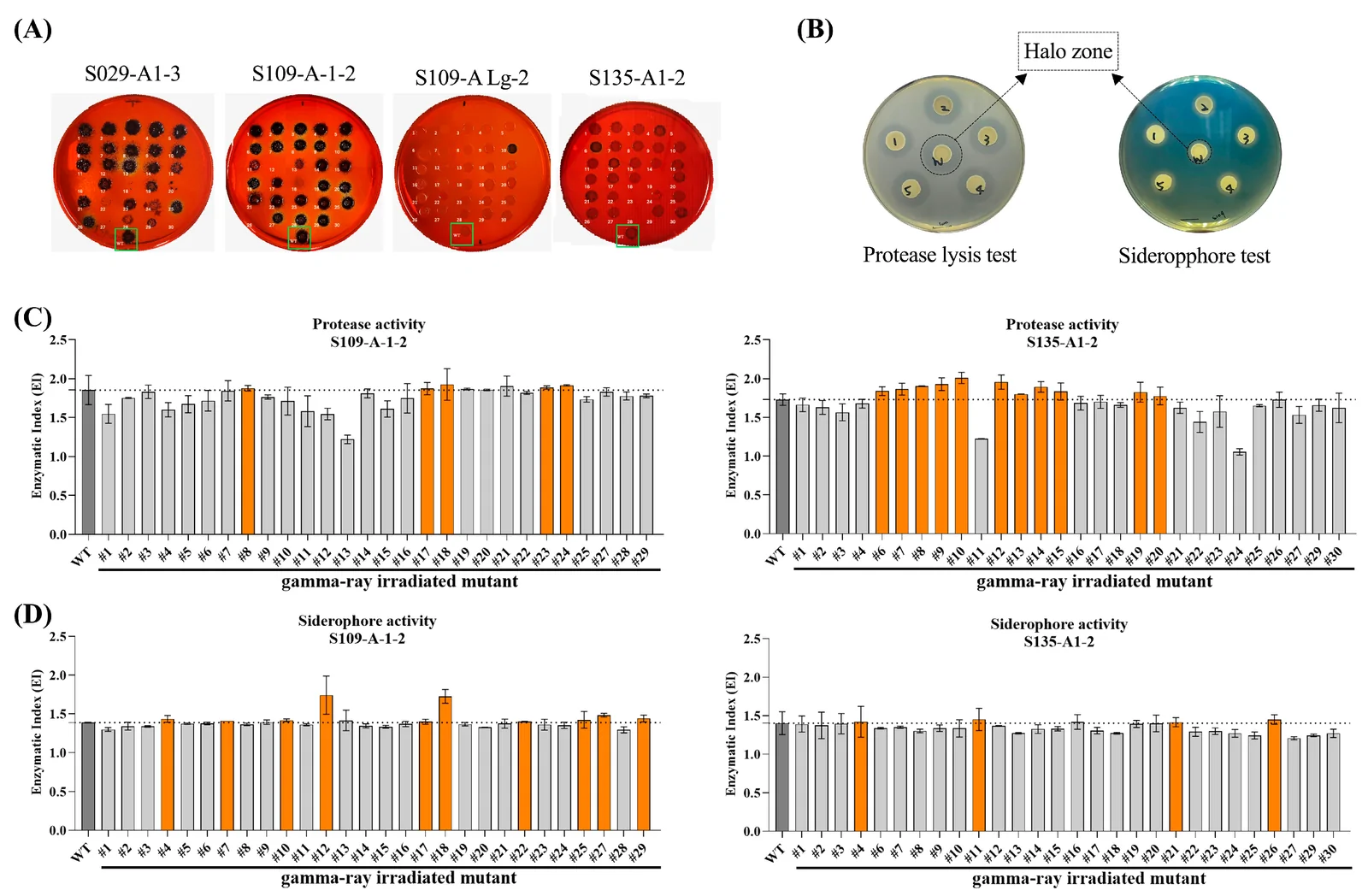
PGPR phenotypes in parental candidates and gamma-irradiated mutants. S029-A1-3, S109-A-1-2, S109-A Lg-2, S135-A1-2, and their derived mutants were examined and quantified for PGPR-related traits, including EPS production (**A**), protease activity (**B**,**C**), and siderophore activity (**B**,**D**). WT: wild-type strains; orange shading indicates protease or siderophore activity higher than the parental strain. The green square presents the EPS production of the parental wild-type strain. The dotted lines indicate the mean values for the wild-type isolates.

**Figure 4:**
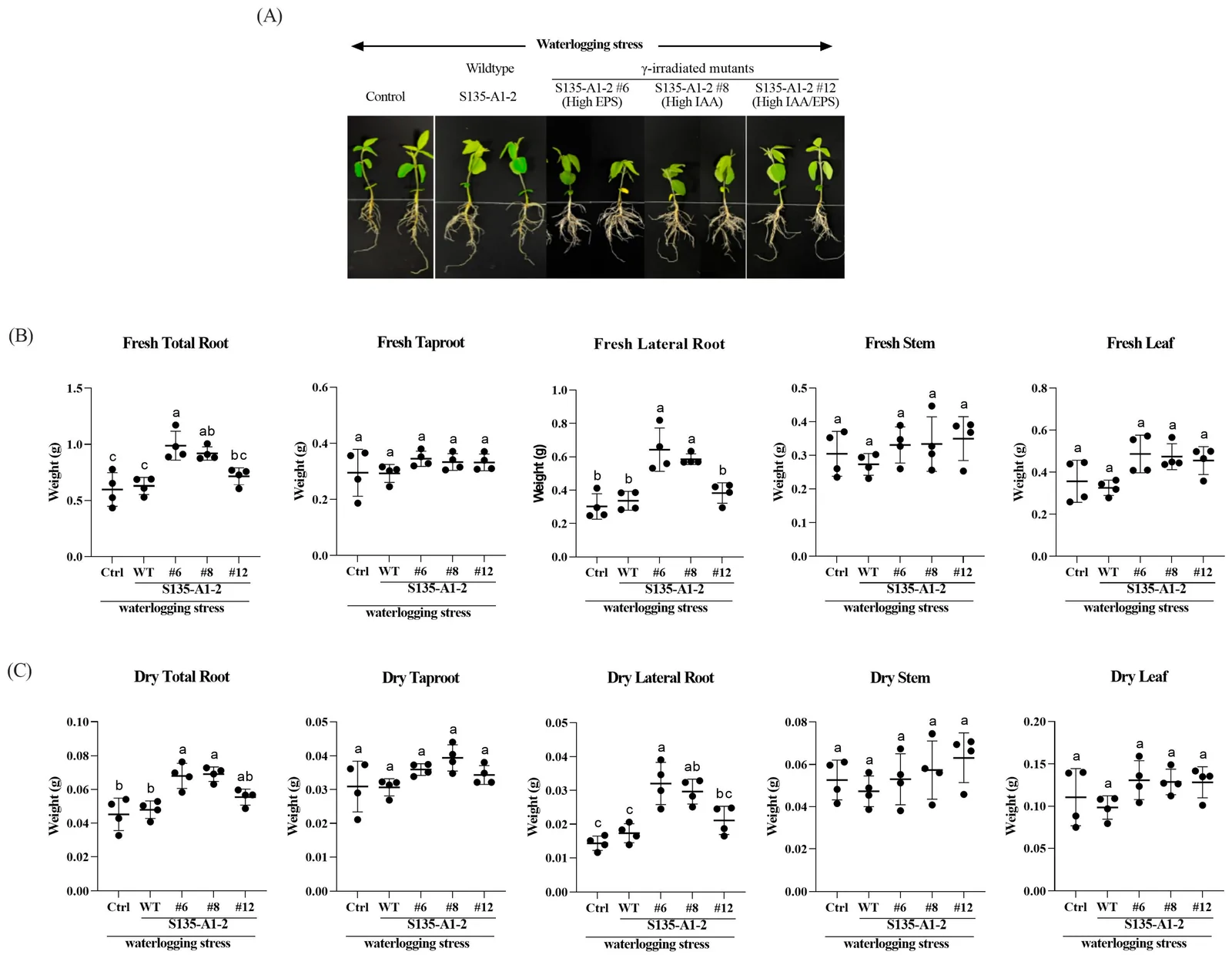
Characteristics of soybean plants inoculated with PGPR under waterlogging stress. Soybean plants were inoculated with *P. megaterium* S135-A1-2 (WT) and mutants #6, #8, and #12 and grown under waterlogging stress for 7 days. (**A**) Plant morphology. (**B**) Fresh weights of total root, taproot, lateral root, stem, and leaf. (**C**) Dry weights of these tissues. Different lowercase letters indicate statistically significant differences among groups determined by one-way ANOVA followed by an appropriate Tukey’s multiple-comparison test (*p* < 0.05). Groups sharing at least one letter are not significantly different.

## Data Availability

The original contributions presented in this study are included in the article/Supplementary Materials. Further inquiries can be directed to the corresponding author.

## Supplementary Materials

The following supporting information can be downloaded at: https://www.mdpi.com/article/10.3390/microorganisms14102145/s1, Figure S1: Characteristics of soybean plants without PGPR under waterlogging stress. Soybean plants were grown for 7 days under waterlogging stress or not. (A) Plant morphology. (B) Fresh weights of total root, taproot, lateral root, and stem. (C) Dry weights of these tissues.

## Abbreviations

The following abbreviations are used in this manuscript:

PGPR	plant growth-promoting rhizobacteria
IAA	Indole-3-acetic acid
EPS	exopolysaccharides
ACC	1-aminocyclopropane-1-carboxylate
