# Supplementary material for: Enhancing the Resistance of Soybean Plants to Waterlogging Stress Using Gamma-Irradiated Priestia megaterium
Source: Microorganisms. 2026 Sep 23;14(10):2145. doi: 10.3390/microorganisms14102145 (PMC13707707; doi:10.3390/microorganisms14102145)
Supplement: Supplementary file 1 [file microorganisms-14-02145-s001.zip › microorganisms-4468811-supplementary.pdf]

Supplement data

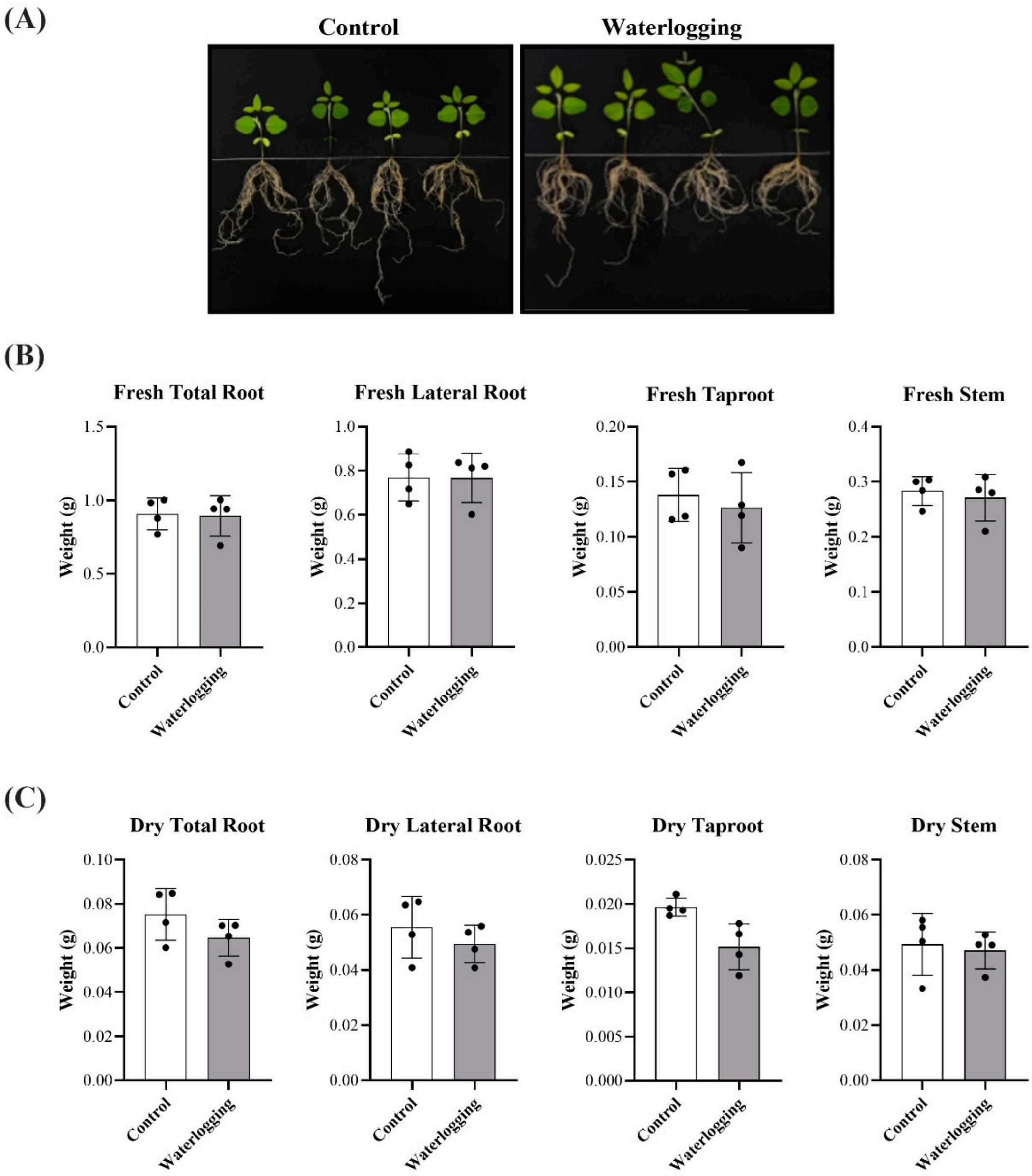

Figure S1. Characteristics of soybean plants without PGPR under waterlogging stress. Soybean plants were grown for 7 days under waterlogging stress or not. (A) Plant morphology. (B) Fresh weights of total root, taproot, lateral root, and stem. (C) Dry weights of these tissues.
